# Composite endpoints, including patient reported outcomes, in rare diseases

**DOI:** 10.1186/s13023-023-02819-x

**Published:** 2023-09-01

**Authors:** Johan Verbeeck, Maya Dirani, Johann W. Bauer, Ralf-Dieter Hilgers, Geert Molenberghs, Rima Nabbout

**Affiliations:** 1 Data Science Institute, Hasselt University, Hasselt, Belgium; 2 reference centre for rare epilepsies Université Paris cité, Assistance Publique-Hôpitaux de Paris, Hôpital Necker-Enfants Malades, Institut Imagine, Paris, France; 3 Department of Dermatology and Allergology, Paracelsus Medical University, Salzburg, Austria; 4 Department of Medical Statistics, MTZ - Medizintechnisches Zentrum, Aachen, Germany; 5 L-Biostat, KULeuven, Leuven, Belgium

**Keywords:** EJP-RD, Epidermolysis bullosa, Generalized pairwise comparisons, Composite endpoints, Quality of life, Rare disease, Patient reported outcomes

## Abstract

**Background:**

When assessing the efficacy of a treatment in any clinical trial, it is recommended by the International Conference on Harmonisation to select a single meaningful endpoint. However, a single endpoint is often not sufficient to reflect the full clinical benefit of a treatment in multifaceted diseases, which is often the case in rare diseases. Therefore, the use of a combination of several clinically meaningful outcomes is preferred. Many methodologies that allow for combining outcomes in a so-called composite endpoint are however limited in a number of ways, not in the least in the number and type of outcomes that can be combined and in the poor small-sample properties. Moreover, patient reported outcomes, such as quality of life, often cannot be integrated in a composite analysis, in spite of their intrinsic value.

**Results:**

Recently, a class of non-parametric generalized pairwise comparisons tests have been proposed, which members do allow for any number and type of outcomes, including patient reported outcomes. The class enjoys good small-sample properties. Moreover, this very flexible class of methods allows for prioritizing the outcomes by clinical severity, allows for matched designs and for adding a threshold of clinical relevance. Our aim is to introduce the generalized pairwise comparison ideas and concepts for rare disease clinical trial analysis, and demonstrate their benefit in a post-hoc analysis of a small-sample trial in epidermolysis bullosa. More precisely, we will include a patient relevant outcome (Quality of life), in a composite endpoint. This publication is part of the European Joint Programme on Rare Diseases (EJP RD) series on innovative methodologies for rare diseases clinical trials, which is based on the webinars presented within the educational activity of EJP RD. This publication covers the webinar topic on composite endpoints in rare diseases and includes participants’ response to a questionnaire on this topic.

**Conclusions:**

Generalized pairwise comparisons is a promising statistical methodology for evaluating any type of composite endpoints in rare disease trials and may allow a better evaluation of therapy efficacy including patients reported outcomes in addition to outcomes related to the diseases signs and symptoms.

**Supplementary Information:**

The online version contains supplementary material available at 10.1186/s13023-023-02819-x.

## Background

### Multiple outcomes in a clinical trial on patients with a rare skin disease

Epidermolysis bullosa simplex (EBS) is a rare, genetic disease, affecting primarily the skin. It is characterized by the formation of blisters under low mechanical stress [1]. While current treatments are limited to alleviation and conventional wound care, a growing number of innovative therapeutic compounds are evaluated in clinical trials. One of these trials was a randomized, placebo-controlled, double-blind, 2-period cross-over phase II/III trial, which assessed the reduction in blisters of an immunomodulatory 1% diacerein cream versus placebo [2]. The 16 paediatric patients, who were randomly assigned to either the placebo or the diacerein treatment, were daily treated for 4 weeks and followed-up for up to 3 months. After a washout period, patients were crossed over to the opposite treatment, following an identical treatment schedule. In each treatment period, blisters in the treated body surface area were counted at the start and the end of the treatment period. The primary endpoint, the proportion of patients with more than 40% reduction in blisters as compared to baseline, was considered more meaningful from a clinical perspective than the raw blister counts. This primary endpoint was tested with a one-sided Barnard test [3], an exact test for a two-by-two table. This test, however, requires separate analyses for each treatment period and led to an inconclusive result [2]. While during the first treatment period 86% of the patients receiving diacerein and 14% of the placebo-treated patients achieved a reduction in blister counts of more than 40% (p = 0.007), during the second period, only 37.5% of the diacerein- treated patients and 17% of the placebo-treated patients achieved a reduction in blister counts of more than 40% (p = 0.32).

**Fig. 1:**
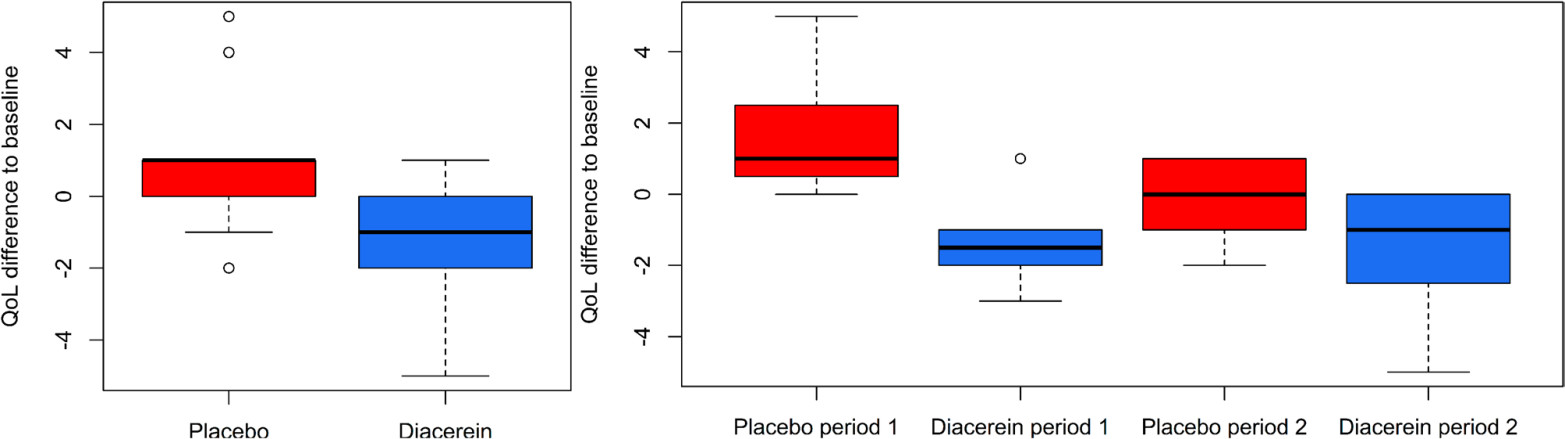
QoL difference between start and end of the treatment in the EBS trial over both treatment periods (left) and by each treatment period (right)

Although the primary endpoint of the EBS trial was based on the blister counts, Quality of Life (QoL) was assessed in addition at the start and end of the treatment period [2] (Fig. 1). A QoL questionnaire assessed by way of 8 questions the hindrance of the disease on daily activities. Given that each question was scored between 0 (no hindrance) and 3 points (very high hindrance), the QoL score ranged from 0 to 24. Because the Barnard test ignores the cross-over design of the study and cannot accommodate the QoL questionnaire, it only uses a fraction of the available information in the cross-over EBS trial. Rather than evaluating a single outcome separately per treatment period, an analysis that uses all information in a single analysis arguably is preferable. This would evade difficulties in interpreting conflicting results from separate analyses of each treatment period. For cross-over trials, such a single small sample test is available [4], but does not allow for assessing multiple outcomes.

### European joint programme on rare diseases

The particular statistical problem of the EBS trial was used as part of an educational webinar, funded by the European Joint Programme on Rare Diseases (EJP RD), EU Horizon 2020 grant no. 825,575. The EJP RD brings together over 130 institutions, including all 24 European Reference Networks (ERN), from 35 countries to create a comprehensive, sustainable ecosystem allowing a virtuous circle between research, care, and medical innovation. The EJP RD has two major objectives. The first one is the improvement of the integration, the efficacy, the production, and the social impact of research in rare diseases through the development, demonstration, and promotion of Europe/world-wide sharing of research, clinical data, materials, processes, knowledge, and know-how. The second objective is to implement and further develop an efficient model of financial support for all types of research on rare diseases (fundamental, clinical, epidemiological, social, economic and health service), coupled with accelerated exploitation of research results for the patients’ benefit. Within the EJP RD, WP20 focuses on accelerating the validation, use, and development of innovative methodologies tailored to clinical trials in rare diseases. One of the integral parts of the WP20 tasks is the advanced webinars that are intended to introduce and disseminate innovative (technical) methodologies for rare disease clinical trials.

The webinar that includes the EBS trial aims to propose statistical methodology for the analysis of composite endpoints in rare diseases, which may include patient relevant outcomes, such as QoL. Registered participants of the webinar were requested to complete a short questionnaire on relevant questions on the topic. The results of this questionnaire, completed by 65 participants (86% not partners of EJP RD), is also presented in this manuscript. The participants consisted of health care professionals, basic researchers, statisticians, patient representatives and health care industry professionals from 22 countries worldwide. Of the respondents, 26 were member of a European reference network.

### Composite endpoint analysis

**Fig. 2:**
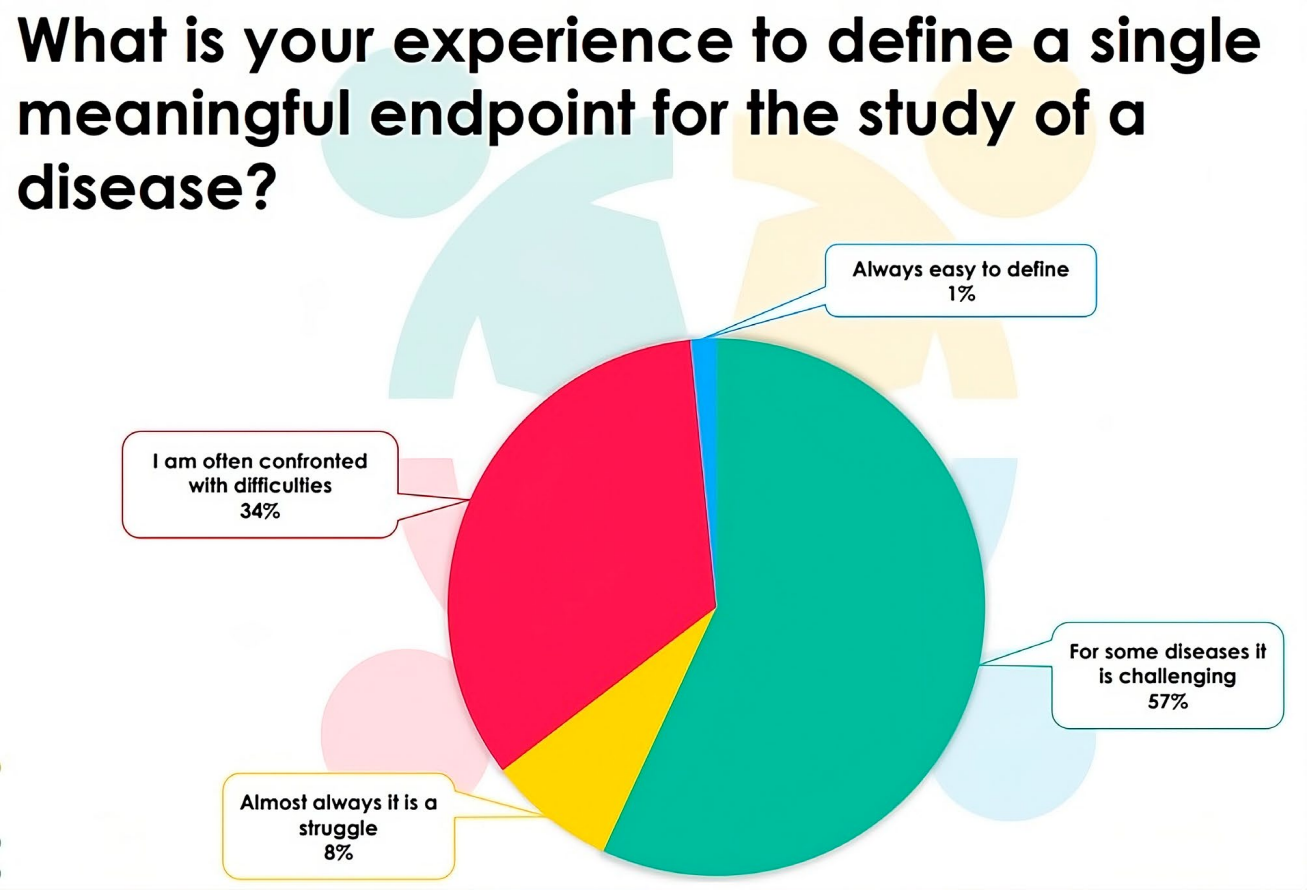
Responses of the participants to the EJP RD webinar (n = 65) to the question: What is your experience to define a single meaningful endpoint for the study of a disease?

**Fig. 3:**
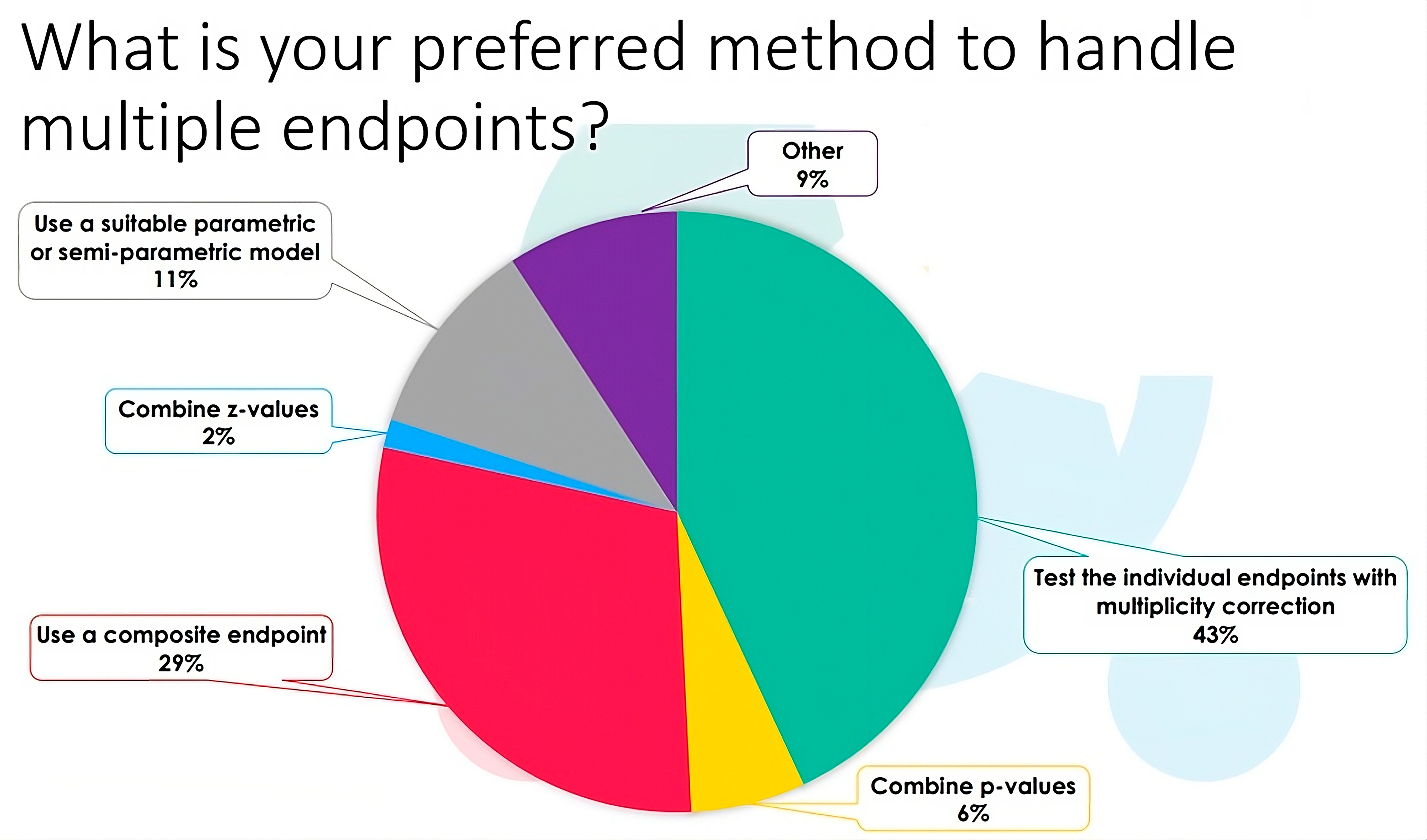
Responses of the participants to the EJP RD webinar (n = 65) to the question: What is your preferred method to handle multiple endpoints?

**Fig. 4:**
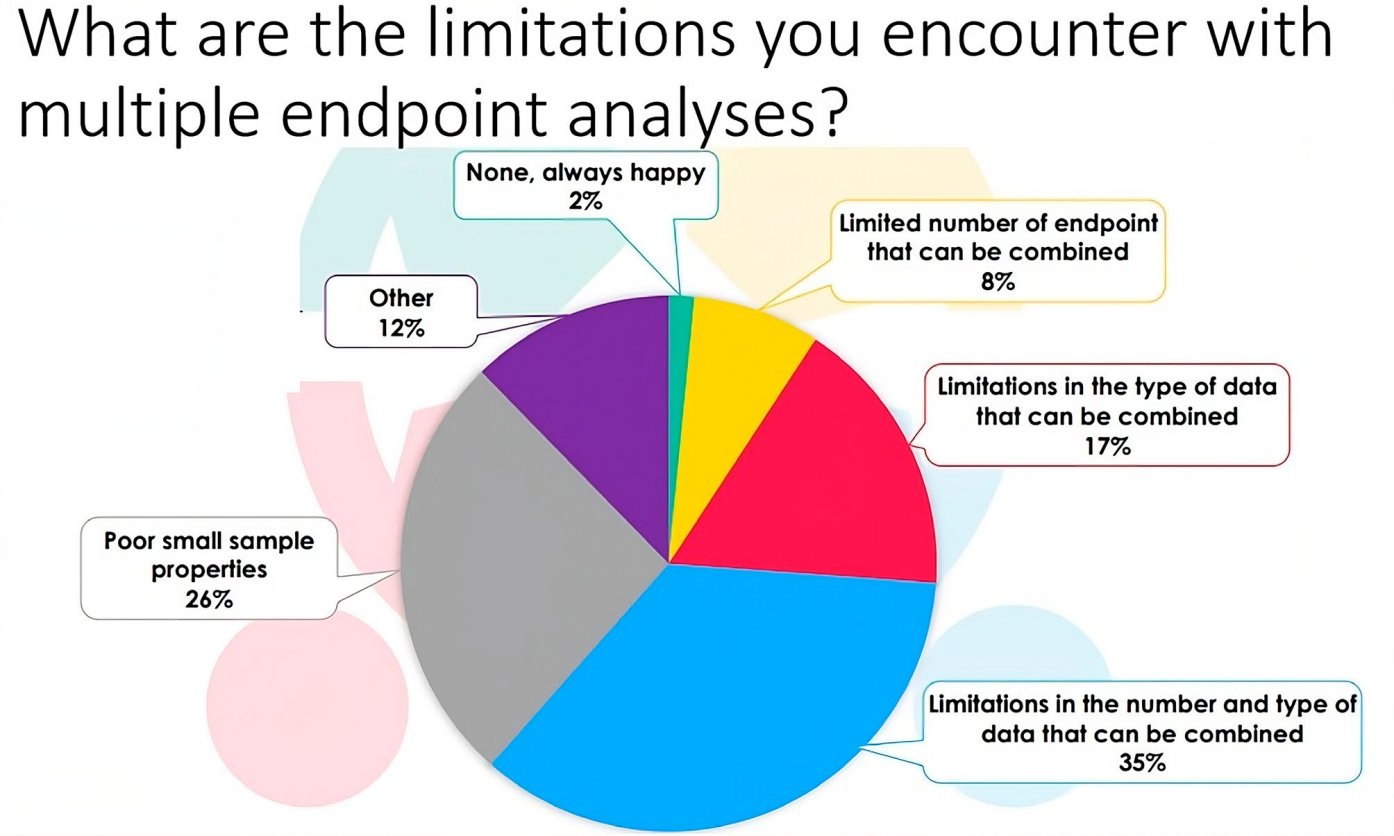
Responses of the participants to the EJP RD webinar (n = 65) to the question: What are the limitations you encounter with multiple endpoint analyses?

The International Conference on Harmonisation recommends selecting a single, clinically meaningful endpoint to assess the efficacy of a treatment in a clinical trial [5]. This meaningful endpoint should be clinically relevant for the disease, be measurable, sensitive to the treatment effect, and ideally be objective [6, 7]. However, in multifaceted diseases, such as is often the case in rare diseases, a single endpoint is frequently not easy to choose or define and is often not sufficient to reflect the full clinical benefit of a treatment. Indeed, among the participants of the webinar, 42% always or often struggle to select or define a single endpoint (Fig. 2). Therefore, a combination of several clinically meaningful outcomes would be very welcome. Multiple outcomes can be combined at several levels: at the level of the subjects (e.g., through clinical indices, ranks, composite endpoints, multivariate parametric, or semi-parametric models); at the level of the test statistics (e.g., combining t-statistics [8, 9] and average z-scores [10]); or at the level of the p-values (e.g., the Lancaster [11] method and its extension to correlated endpoints [12] or multiple testing procedures [13]). While the majority of the respondents (43%) prefer to test the individual endpoints separately and correct for multiple testing (Fig. 3), this approach implies that the required significance level is substantially smaller than 5%, making it more difficult to detect a treatment effect, especially in small samples. By combining the outcomes, a multiplicity correction for testing each outcome individually is avoided, which will, in general, lead to an increased power to detect a treatment effect and thus reduction in sample size, although the ability to test individual outcomes is lost [14]. Many of the methodologies that combine clinical outcomes, however, have limitations. They either ignore the correlation between the outcomes, are limited to a certain type of data, have no straightforward effect size measure to quantify the effect of the treatment, or the small sample properties of these tests are underwhelming. The two most important limitations the respondents of the EJP RD webinar identify are the limitations in the number and type of data that can be combined (35%) and the poor small sample properties (26%) (Fig. 4). While the power to detect a treatment difference by parametric and semi-parametric methods is often superior compared to the non-parametric methods, they may be limited in the number and type of endpoints that can be combined and may be less adequate for small-sample trials [14]. The non-parametric methods, on the other hand, are less restricted and often have good small-sample properties. Especially the generalized pairwise comparisons method has recently attracted considerable attention, not in the least because this very flexible class of methods allows prioritizing the outcomes by clinical severity.

### Generalized pairwise comparisons (GPC)

**Fig. 5:**
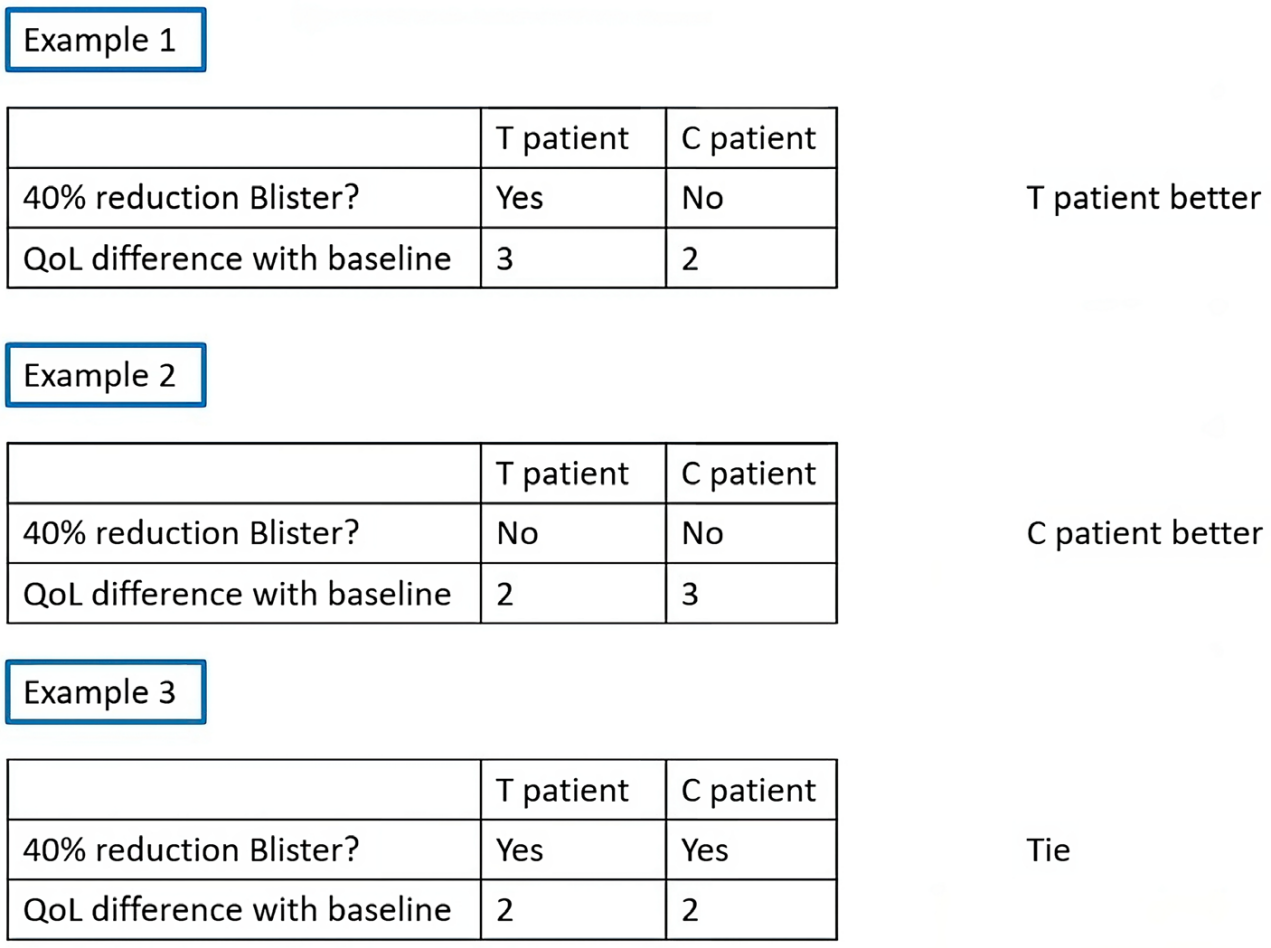
Three examples of pairwise comparisons of a composite prioritized GPC, with 40% reduction in blister prioritized over the difference in QoL. The pairwise comparison is initiated on the blister outcome. If a subject with a better outcome is assigned (example 1) the QoL outcome is not evaluated. Only when the better outcome cannot be decided on the blister outcome (example 2 and 3) the QoL outcome is compared. If both subjects have equal values in each outcome, the pair is considered a tie (example 3). T= Experimental treatment and C= Control treatment

Generalized pairwise comparisons offer a very flexible class of statistical methodologies, proposed for the analysis of multiple outcomes in a two-arm clinical trial [15–18]. The underlying principle of the methodology is pairwise comparisons, as in the alternative version of the Mann-Whitney test [19]. In fact, with a single outcome, the GPC analysis equals the non-parametric Mann-Whitney test. GPC allows combining any number and type of outcomes and the most frequently used GPC test allows prioritizing these outcomes by clinical severity. Briefly, all possible pairs of subjects are formed with one subject from each treatment arm. Within each pair, it is decided which of the subjects has the better outcome of the highest priority. If the better outcome cannot be decided on the outcome of highest priority, the comparison moves to the next outcome in the priority list and continues until a better outcome can be declared, or until the last outcome results in no assignment of a better outcome. In the latter case, there is a tie (Fig. 5). The definition of a better outcome is determined a priori per outcome and may depend on a threshold [16]. For example, a subject has a more favorable outcome compared to another, only if the difference in number of blisters is more than, for example, three, or any other threshold considered clinically relevant. Although GPC has been applied in other clinical areas, the largest number of applications are seen in cardiology, in post-hoc analyses [20–23], in the design of clinical trials [24, 25], as well as in primary endpoint analyses [26–30].

Several hypothesis tests for detecting a treatment difference have been proposed for GPC [31]. While most tests are suitable only for sufficiently large-sample trials, one test, the exact permutation test, has good small-sample properties too [31, 32]. In a two-arm trial with only 5 subjects in each treatment arm, the exact permutation test maintains the nominal significance level, a requirement for the validity of a test. The size of the treatment effect in GPC can be expressed by the so-called net treatment benefit, which is a value between − 1 and 1 and corresponds to the difference in probability that a random subject in the experimental arm is doing better than a random subject in the control arm. Positive values indicate a beneficial treatment effect, while negative values reflect harm. The net treatment benefit can also be transformed to a ratio, called the success or win odds [33–35], where values above 1 indicate a beneficial treatment effect and values below 1 harm. Next to the net treatment benefit and its transformations, also the win ratio [17] has been suggested as a treatment effect measure. It has, however, been criticized to ignore the tied pairs and to overestimate the treatment effect [34–36].

Besides the prioritized GPC, several variations of the algorithm exist. The non-prioritized GPC evaluates each of the outcomes in all pairs [18, 37], while the matched GPC only compares the outcomes in a subset of the pairs that are matched by design of the trial [38] or by risk [17]. The latter GPC variant uses a different hypothesis test in small samples, which requires at least 20 subjects to maintain the nominal significance level [39, 40]. Although in the EBS trial, a matched comparison seems natural given the cross-over design, it has been shown that in certain situations, such as in the GPC test, ignoring the matching still leads to asymptotically valid results [41]. While missing data are handled naturally in a prioritized GPC, by moving to the next outcome in the priority list, the matched GPC requires fully observed outcomes in both treatment periods.

Hence, GPC resolves many issues of composite endpoint analyses for small samples, following the composite endpoint definition of McLeod et al. [42]. GPC allows for any number and type of outcomes, allows for priority ranking of outcomes by clinical severity, has straightforward measures to quantify the effect of the treatment, has good small-sample properties, and captures correlation between the outcomes [18]. Moreover, GPC has been accepted as a primary endpoint analysis for the approval of the drug tafamidis in the rare disease amyloid cardiomyopathy by both regulatory authorities FDA and EMA [27].

## Aims and methodology

**Fig. 6:**
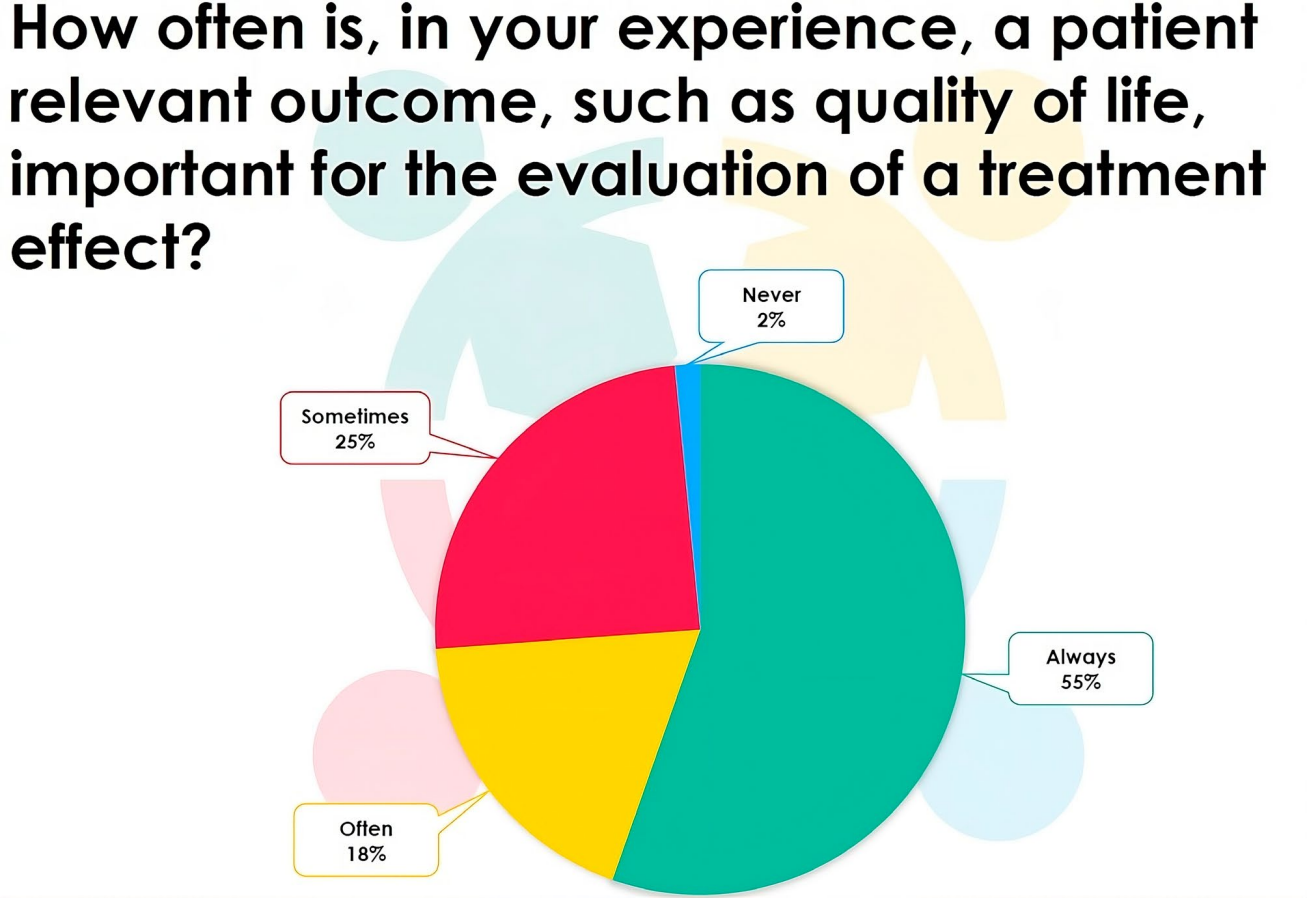
Responses of the participants to the EJP RD webinar (n = 65) to the question: How often is, in your experience, a patient relevant outcome, such as quality of life, important for the evaluation of a treatment effect?

As an illustration of the GPC methodology, we re-analyze the EBS trial by including the QoL outcome to the blister outcome. Although often ignored, clinically, it is sensible to evaluate how treatment affects QoL. Indeed 55% of the participants to the EJP RD webinar indicate that QoL is always important in the evaluation of a treatment (Fig. 6). We analyze the composite endpoint with both a prioritized, non-prioritized and matched GPC test and demonstrate in a simulation study, based on the EBS trial, the power of GPC to detect a treatment effect and evaluate its validity in small samples. In the prioritized GPC, the blisters are ranked as more important than the QoL. Since some subjects have missing data, only 13 subjects can be used in the matched GPC analysis.

In the simulation study the blister count and QoL measurements for each subject in the EBS trial are permuted 5000 times, meaning that the original treatment allocation is allowed to change per subject and permuted sample. The permutation ensures that any treatment effect present in the data is removed, which allows the evaluation of the type I error. To evaluate the power of the GPC test to detect a treatment effect if there is one, an effect is added in the permuted samples for both the blisters and the QoL outcome, by randomly sampling from a mean 3 Poisson distribution. These simulated treatment effects, which were considered realistic by clinicians, correspond to a higher blister count in placebo subjects and more daily hindrance.

## Results

The simulation study shows that the prioritized (considering the blister reduction more important than the QoL) and non-prioritized GPC (considering the blister reduction and QoL equally important) one- and two-sided test is valid in small samples, given that the type I error is well controlled (i.e., it is close to 5%) (Table 1). The single analysis of the blister counts shows a liberal two-sided test, which may be partly due to the large number of ties that are present when comparing whether a subject had a 40% reduction in blisters in the pairs. As anticipated, the type I error in the matched GPC is not controlled in a sample with less than 20 subjects (Table 1).

When adding the QoL outcome to the blister outcome, the power to detect a treatment effect increases from 59% to more than 90% for both the prioritized and non-prioritized GPC. This shows that finding a treatment effect when re-analyzing the EBS trial is not a coincidence, but will occur more than 90% if a treatment effect similar in size to the one simulated is present. Even though the matched GPC is conservative, it still shows a power of almost 60% to detect a treatment effect (Table 1).

**Table 1:** Two-sided (one-sided) type I error and power of the single and composite GPC analyses in 5000 permuted samples of the original EBS trial. The blister outcome indicates if a subject has a 40% reduction in blisters

	Type I error	Power
	**Unmatched GPC**	
Single blister	0.0692 (0.0216)	0.5904 (0.7202)
Single QoL	0.0514 (0.0486)	0.8642 (0.9302)
Multi Prioritized	0.0514 (0.0510)	0.9594 (0.9812)
Multi Non-prioritized	0.0490 (0.0524)	0.9886 (0.9716)
	**Matched GPC**	
Single Blister	0.0348 (0.0632)	0.4751 (0.6029)
Single QoL	0.0422 (0.0610)	0.7044 (0.8650)
Multi Prioritized	0.0260 (0.0258)	0.5824 (0.8210)

When re-analyzing the EBS trial with an unmatched GPC for both the blister count and QoL outcome separately (Table 2), which equals the Mann-Whitney test, there is insufficient evidence that diacerein changes the fraction of subjects with a 40% reduction in blisters (p = 0.0701), but there is evidence that diacerein improves QoL (p = 0.0019). When adding both outcomes together in a composite GPC analysis, there is evidence for a positive treatment effect of diacerein, both when prioritizing the outcomes (p = 0.0051), treating the outcomes as equally important (p = 0.0022) and in a matched analysis (p = 0.0209). The net treatment benefit, or the net probability of a better outcome for a subject treated with diacerein compared to a subject treated with standard of care, is 59% (95% CI: 19–82%) with prioritized outcomes, 48% (95% CI:21–68%) when treating blisters and QoL as equally important and 62% (95% CI: 9–88%) in a matched analysis (Table 2).

The net treatment benefit, in contrast to the other GPC statistics, conveniently allows to gauge the contribution of each outcome to the overall effect. For the prioritized GPC, for example, 55% of the pairs were decided on the blister outcome, compared to 38% for the QoL (Table 2).

**Table 2:** Original EBS trial data analysis of the composite blister and QoL outcomes with the prioritized, non-prioritized and matched GPC. The blister outcome indicates if a subject has a 40% reduction in blisters. NTB = Net Treatment Benefit, CI = Confidence Interval

	# wins	#losses	#ties	NTB (95%CI)	p-value two-sided
	**Prioritized GPC**				
Blister	99 (44%)	24 (11%)		0.33	
QoL	72 (32%)	14 (6%)		0.26	
Total	171 (76%)	38 (17%)	16 (7%)	0.59 (0.19;0.82)	0.0051
	**Non-prioritized GPC**				
Blister	99 (44%)	24 (11%)	102 (45%)	0.33	0.0701
QoL	162 (72%)	22 (10%)	41 (18%)	0.62	0.0019
Total				0.48 (0.21;0.68)	0.0022
	**Matched GPC**				
Blister	5 (38%)	1 (15%)		0.23	
QoL	5 (38%)	0 (0%)		0.20	
Total	10 (77%)	2 (15%)	1 (8%)	0.62 (0.09;0.88)	0.0209

To gain further insights into the treatment effect, the prioritized and non-prioritized GPC analysis can be repeated for each treatment period separately. Both the prioritized and non-prioritized GPC show that the treatment effect is mainly present in the first treatment period (Table 3). A matched GPC can obviously not be split into a per treatment period analysis, since it compares the outcomes between 2 treatment periods within a subject.

**Table 3:** Original EBS trial data analysis of the composite blister and QoL outcome with the prioritized and non-prioritized split per treatment period. The blister outcome indicates if a subject has a 40% reduction in blisters. NTB = Net Treatment Benefit, CI = Confidence Interval

	# wins	#losses	#ties	NTB (95%CI)	p-value two-sided
	**Treatment period 1**				
	**Prioritized GPC**				
Blister	30 (54%)	3 (5%)		0.48	
QoL	17 (30%)	0 (0%)		0.30	
Total	47 (84%)	3 (5%)	6 (11%)	0.79 (0.21;0.96)	0.0077
	**Non-prioritized GPC**				
Blister	30 (53%)	3 (5%)	23 (41%)	0.48	0.0662
QoL	43 (77%)	2 (4%)	11 (20%)	0.73	0.0076
Total				0.61 (0.18;0.84)	0.0134
	**Treatment period 2**				
	**Prioritized GPC**				
Blister	18 (32%)	5 (9%)		0.23	
QoL	16 (29%)	9 (16%)		0.13	
Total	34 (61%)	14 (25%)	8 (14%)	0.36 (-0.24;0.76)	0.2368
	**Non-prioritized GPC**				
Blister	18 (32%)	5 (9%)	33 (49%)	0.23	0.0701
QoL	36 (64%)	11 (20%)	9 (16%)	0.45	0.0019
Total				0.34 (-0.05;0.64)	0.1073

## Discussion

In multifaceted diseases, such as is often the case in rare diseases, a single endpoint is often not sufficient to reflect the full clinical benefit of a treatment. We have shown the usefulness of a fairly recent non-parametric statistical methodology, called generalized pairwise comparison, for the analysis of composite endpoints in rare diseases. More specifically, GPC is a very flexible tool that allows for the combination of any type and number of outcomes, including patient relevant outcomes, and has very good small-sample properties. The need for such a method was supported by the questionnaire responses of participants to a recent EJP RD webinar on rare disease, which revealed that selecting a single outcome as an endpoint in a clinical trial in rare disease is often difficult, that patient reported outcomes, such as quality of life are important outcomes in the evaluation of a treatment, but that current methodology is limited in its ability to combine patient reported outcomes with more traditional outcomes.

The main advantage of selecting and combining any outcome as an endpoint in a clinical trial, is the great flexibility in describing the clinical benefit of a treatment, especially in multifaceted diseases. Additionally, composite endpoints may have an increased power to detect a treatment effect, compared to a single outcome, which is relevant and important in small-sample trials. However, when more than one outcome composes a clinical endpoint, some outcomes may be clinically more important than others. While many methodologies do not allow for prioritizing outcomes, a clinical hierarchy in outcomes is naturally embedded in a prioritized GPC. When no clinical priority is present or wanted among the outcomes, the outcomes can be treated equally important in a non-prioritized GPC. The choice between prioritized and non-prioritized is determined by the clinical setting and appropriateness and should be decided on a trial-by-trial basis in a discussion among statisticians, clinicians and patients. The non-prioritized GPC has the additional benefit that individual outcomes can still be tested, as demonstrated in Tables 1 and 2. A hierarchical testing procedure can be adopted by testing the overall effect first, followed by the individual outcomes, potentially prioritized. Although GPC variants for matched designs exist, their application is limited by a sample size of 20 subjects.

Additional benefits of the GPC methodology are the straightforward interpretation of the treatment effect measure, such as the net treatment benefit, which also gives insight into the partial contribution of each outcome to the overall test. Although not explicitly modelled, the correlation between the outcomes in a composite endpoint is captured by both the prioritized and non-prioritized GPC, albeit differently [18].

The flexibility of GPC additionally allows one-sided hypotheses tests, which result in a gain in power, and allows for defining a threshold of clinical relevance in the pairwise comparisons. For example, the blisters in the EBS trial were re-analyzed with the original endpoint of a 40% reduction in blisters. However, dichotomizing the number of blisters may lead to a considerable number of ties in the pairwise comparisons and hence loss of information. On the other hand, evaluating the treatment effect on the raw blister counts is surrounded by uncertainty, since blisters may appear and disappear spontaneously in EBS patients. A threshold may decrease the effect of this uncertainty on the results by considering, for example, only a difference of at least 3 blisters between two patients as a better outcome. It is important to note that the amount of ties increases, when going from the raw counts to the counts with a threshold and to a dichotomization of the counts. Which means that with more ties, more information from the outcomes with a lower priority is used in a prioritized GPC. For example, if we re-analyze the EBS trial with a prioritized composite endpoint composed by the raw blister counts (or rather the standardized difference in blisters) and QoL improvement, less information is used from the QoL outcome (2% of the pairs) compared to the composite endpoint with the dichotomized counts (38% of the pairs) (Appendix Tables 1 and 2).

The re-analysis of the EBS trial with all GPC variants on the composite endpoint, composed of the 40% in blisters and QoL improvement, show evidence of a treatment effect of the diacerein cream. Additionally, the analysis per treatment period demonstrates that the treatment effect is mainly present in the first treatment period. Potentially, the effect in the second treatment period is influenced by a cross-over effect, as less baseline blisters were observed at the start of the second treatment period compared to the first [2]. Indeed, measurements of the blisters at the 3 month follow-up period after the first treatment period indicate a persisting treatment effect [2].

GPC has been applied mainly in the cardiovascular clinical area for the re-analysis or the design of large sample clinical trials. In the presence of survival outcomes only, a GPC allows a clinically more sensible interpretation compared to a time-to-first event analysis [17, 22, 23, 26, 29, 30]. Additionally, continuous outcomes, such as a 6-minute walk test (6MWT) or categorical outcomes, such as QoL, have been added to survival outcomes in GPC endpoints in cardiovascular trials [18, 23, 24, 28]. In oncology, GPC re-analyses have been applied for benefit-risk assessments [43]. Specifically in the rare disease domain, a GPC re-analysis of the randomized, double-blind, phase 3 COMET trial, prioritizing the primary (forced vital capacity) and secondary outcome (6MWT), provided evidence of efficacy of avalglucosidase alfa therapy (n = 51) over alglucosidase alfa (n = 49) in Pompe disease, while the original analysis failed to show superiority on the primary endpoint (forced vital capacity) [44]. Moreover, in the double-blind, placebo-controlled, phase 3 ATTR-ACT trial, the primary GPC analysis, prioritizing time to death followed by time to hospitalization, showed evidence of efficacy of tafamidis (n = 264) over placebo (n = 177) and lead to drug approval in transthyretin amyloid cardiomyopathy patients [27].

The EJP RD webinar introduced the GPC methodology for composite endpoints in rare diseases, but the value of this method should be further investigated by comparing it to other methods, such as, but not limited to, parametric combined models [45] with split sample [46–48] or pseudolikelihood inference [47–49], the non-parametric O’Brien ordinary and general least square methods [8] and its improved version by Läuter [9] and randomization based inference [50] on a permutation test. Although the influence of missing data [51] and corrections for censored data [52] have been proposed in GPC, further investigation is required, specifically in small sample size trials. Finally, non-parametric statistical methods typically only allow for covariate adjustment through stratification [53]. In rare disease clinical trials, however, where the sample size is already small, dividing the trial sample in even smaller strata may not be feasible. Interestingly, the GPC statistics on a single outcome can be incorporated in a semi-parametric modelling framework, which allows for the correction of multiple covariates [54, 55]. Further research is required to extend these models to composite endpoints and evaluate its performance in small samples.

Statistical programs for the exact permutation hypothesis test for GPC are available in SAS and R [32].

## Conclusions

Generalized pairwise comparisons (GPC) is a promising statistical methodology for evaluating any type of composite endpoints in rare disease trials and may allow a better evaluation of therapy efficacy including patients reported outcomes in addition to outcomes related to the diseases signs and symptoms achieving easily what is recommended for the clinical outcome assessment.

## Appendix

**Table 1:** Two-sided (one-sided) type I error and power of the single and composite GPC analyses in 5000 permuted samples of the original EBS trial. The blister outcome is treated as a standardized difference of the number of blisters $\left(\frac{\text{N}\text{u}\text{m}\text{b}\text{e}\text{r } \text{o}\text{f } \text{b}\text{l}\text{i}\text{s}\text{t}\text{e}\text{r}\text{s } \text{a}\text{t } \text{b}\text{a}\text{s}\text{e}\text{l}\text{i}\text{n}\text{e}-\text{N}\text{u}\text{m}\text{b}\text{e}\text{r } \text{o}\text{f } \text{b}\text{l}\text{i}\text{s}\text{t}\text{e}\text{r}\text{s } \text{a}\text{t } \text{w}\text{e}\text{e}\text{k } 4}{\text{N}\text{u}\text{m}\text{b}\text{e}\text{r } \text{o}\text{f } \text{b}\text{l}\text{i}\text{s}\text{t}\text{e}\text{r}\text{s } \text{a}\text{t } \text{b}\text{a}\text{s}\text{e}\text{l}\text{i}\text{n}\text{e}}\right)$

	Type I error	Power
	**Unmatched GPC**	
Single blister	0.0438 (0.0450)	0.5138 (0.6650)
Single QoL	0.0490 (0.0528)	0.7940 (0.8888)
Multi Prioritized	0.0442 (0.0458)	0.5402 (0.6852)
Multi Non-prioritized	0.0510 (0.0502)	0.9250 (0.9670)
	**Matched GPC**	
Single Blister	0.0472 (0.0620)	0.2784 (0.5136)
Single QoL	0.0414 (0.0540)	0.6536 (0.8068)
Multi Prioritized	0.0414 (0.0724)	0.2714 (0.5440)

**Table 2:** Original EBS trial data analysis of the composite blister and QoL outcomes with the prioritized, non-prioritized and matched GPC. The blister outcome is treated as a standardized difference of the number of blisters $\left(\frac{\text{N}\text{u}\text{m}\text{b}\text{e}\text{r } \text{o}\text{f } \text{b}\text{l}\text{i}\text{s}\text{t}\text{e}\text{r}\text{s } \text{a}\text{t } \text{b}\text{a}\text{s}\text{e}\text{l}\text{i}\text{n}\text{e}-\text{N}\text{u}\text{m}\text{b}\text{e}\text{r } \text{o}\text{f } \text{b}\text{l}\text{i}\text{s}\text{t}\text{e}\text{r}\text{s } \text{a}\text{t } \text{w}\text{e}\text{e}\text{k } 4}{\text{N}\text{u}\text{m}\text{b}\text{e}\text{r } \text{o}\text{f } \text{b}\text{l}\text{i}\text{s}\text{t}\text{e}\text{r}\text{s } \text{a}\text{t } \text{b}\text{a}\text{s}\text{e}\text{l}\text{i}\text{n}\text{e}}\right)$. NTB = Net Treatment Benefit, CI = Confidence Interval

	# wins	#losses	#ties	NTB (95%CI)	p-value two-sided
	Prioritized GPC				
Blister	130 (66%)	61 (31%)		0.35	
QoL	4 (2%)	0 (0%)		0.02	
Total	134 (68%)	61 (31%)	1 (0.5%)	0.37 (-0.06;0.81)	0.0935
	**Non-prioritized GPC**				
Blister	130 (66%)	61 (31%)	5 (3%)	0.35	0.1124
QoL	141 (72%)	19 (10%)	36 (18%)	0.62	0.0027
Total				0.49 (0.15;0.83)	0.0049
	**Matched GPC**				
Blister	5 (42%)	5 (42%)		0.00	
QoL	2 (17%)	0 (0%)		0.20	
Total	7 (58%)	5 (42%)	0 (0%)	0.17 (-0.36;0.61)	0.5637

### Electronic supplementary material

Below is the link to the electronic supplementary material.

Supplementary Material 1

Supplementary Material 2

## Data Availability

The data that supports the findings in this paper are available within the paper and its Supplementary Information.

## Abbreviations

CI: Confidence Interval
EBS: Epidermolysis bullosa simplex
EJP RD: European Joint Programme on Rare Diseases EMA = European Medicine Agency
ERN: European Reference Networks EU = Europe
FDA: Food and Drug Administration GPC = Generalized Pairwise Comparisons NTB = Net Treatment Benefit
WP20: Workpackage 20 QoL = Quality of Life
